# Evidence for disruption of diurnal salivary cortisol rhythm in childhood obesity: relationships with anthropometry, puberty and physical activity

**DOI:** 10.1186/s12887-020-02274-8

**Published:** 2020-08-12

**Authors:** Ting Yu, Wei Zhou, Su Wu, Qianqi Liu, Xiaonan Li

**Affiliations:** 1 Department of Child Health Care, Children’s Hospital of Nanjing Medical University, 72 Guangzhou Road, Nanjing, 210008 China; 2 Department of Endocrinology, Children’s Hospital of Nanjing Medical University, Nanjing, 210008 China; 3 Institute of Pediatric Research, Nanjing Medical University, Nanjing, China

**Keywords:** Cortisol, Circadian rhythm, Childhood obesity, Puberty, Physical activity

## Abstract

**Background:**

The aim of this study was to examine the characteristics of diurnal cortisol rhythm in childhood obesity and its relationships with anthropometry, pubertal stage and physical activity.

**Methods:**

Thirty-five children with obesity (median age: 11.80[interquartile range 10.30, 13.30] and median BMI z-score: 3.21[interquartile range 2.69, 3.71]) and 22 children with normal weight (median age: 10.85[interquartile range 8.98, 12.13] and median BMI z-score: − 0.27[interquartile range − 0.88, 0.35]) were recruited. Saliva samples were collected at 08:00, 16:00 and 23:00 h. Cortisol concentrations at 3 time points, corresponding areas under the curve (AUCs) and diurnal cortisol slope (DCS) were compared between the two groups. Anthropometric measures and pubertal stage were evaluated, and behavioural information was obtained via questionnaires.

**Results:**

Children with obesity displayed significantly lower cortisol_08:00_ (median [interquartile range]: 5.79[3.42,7.73] vs. 8.44[5.56,9.59] nmol/L, *P* = 0.030) and higher cortisol_23:00_ (median [interquartile range]: 1.10[0.48,1.46] vs. 0.40[0.21,0.61] nmol/L, *P* < 0.001) with a flatter DCS (median [interquartile range]: − 0.29[− 0.49, 0.14] vs. -0.52[− 0.63, 0.34] nmol/L/h, *P* = 0.006) than their normal weight counterparts. The AUC increased with pubertal development (AUC_08:00–16:00_:*P* = 0.008; AUC_08:00–23:00_: *P* = 0.005). Furthermore, cortisol_08:00_ was inversely associated with BMI z-score (β = − 0.247, *P* = 0.036) and waist-to-height ratio (WHtR) (β = − 0.295, *P* = 0.027). Cortisol_23:00_ was positively associated with BMI z-score (β = 0.490, *P*<0.001), WHtR (β = 0.485, *P*<0.001) and fat mass percentage (FM%) (β = 0.464, *P*<0.001). Absolute values of DCS were inversely associated with BMI z-score (β = − 0.350, *P* = 0.009), WHtR (β = − 0.384, *P* = 0.004) and FM% (β = − 0.322, *P* = 0.019). In multivariate analyses adjusted for pubertal stage and BMI z-score, Cortisol_08:00_, AUC_08:00–16:00_ and absolute values of DCS were inversely associated with the relative time spent in moderate to vigorous intensity physical activity (*P* < 0.05). AUC_16:00–23:00_ was positively associated with relative non-screen sedentary time and negatively associated with sleep (*P* < 0.05).

**Conclusions:**

The disorder of diurnal salivary cortisol rhythm is associated with childhood obesity, which is also influenced by puberty development and physical activity. Thus, stabilizing circadian cortisol rhythms may be an important approach for childhood obesity.

## Background

Accompanied by economic development and lifestyle changes, the prevalence of childhood obesity has increased rapidly worldwide, leading to obesity-related metabolic diseases in adulthood, such as non-alcoholic fatty liver disease, type 2 diabetes, and cardiovascular disease [1, 2]. Indeed, the unhealthy lifestyle and academic demands of children are increasingly interfering with biological rhythms, which might contribute to childhood obesity and negative health outcomes [3, 4]. Therefore, it is essential to identify novel contributors to the underlying physiology of childhood obesity.

Cortisol is a primary product of the hypothalamic-pituitary-adrenal (HPA) axis and acts as the terminal effector of this axis on other systems [5]. In both human and animal models, cortisol has been causally demonstrated to promote fat accumulation and weight gain as well as glucose homeostasis and lipid metabolism [6, 7]. Considering that the production, secretion and abundance of cortisol are regulated in a robust time-of-day-dependent manner [8], the diurnal cortisol rhythm is a good indicator for comprehensive evaluation of HPA axis activity. Cortisol rhythms are believed to be established between 2 and 9 months in early life [9], as mediated by a combination of influences such as the light-dark cycle, pubertal development, feeding, sleep, and physical activity [10]. Under non-stress conditions, the secretion and release of cortisol follows a typical circadian rhythm: cortisol rapidly increases 30 to 40 min after awakening, followed by a sharp decline during the next few hours and a gradually decline during the remainder of the day until reaching the lowest level at midnight [11, 12].

An interaction between the HPA axis and obesity has long been proposed. On the one hand, cortisol controls body weight via effects on both food intake and energy expenditure as well as adipogenic pathways in abdominal adipose tissue [13]. On the other hand, obesity constitutes a chronic stressor and in turn alters the activity of the stress axis [14]. Recently, it has been proposed that obesity is associated with circadian disruption and often accompanied by altered HPA axis rhythmicity [5]. Adults with obesity usually display blunted diurnal HPA axis functioning, which manifests as decreased cortisol variability, lower morning levels, or a smaller change in cortisol throughout the day [15–17]. However, studies of diurnal cortisol patterns in childhood obesity have resulted in different findings. For example, Kjolhede reported that average salivary cortisol levels throughout the day were significantly lower in children with obesity [18], whereas Hillman showed that with an increasing degree of adiposity in adolescent girls, there may be reduced serum cortisol levels during the day and increased levels at night [14]. Conversely, another study reported no significant association between the HPA axis and percent body fat in pre-pubertal children with obesity [19]. A potential explanation for these variable findings is with regard to methodological differences such as different measurement methods (e.g., enzyme immunoassay, radioimmunoassay, chemiluminescence immunoassay) and sampling time. Considering the characteristics of children’s growth, regulation of the HPA axis in children may be affected by more complex factors than those in adults, such as age, pubertal development and stress-related activities (dietary consumption, physical activities, etc.). Therefore, exploring the factors influencing children’s HPA axis may help in reaching a complete understanding of the links between dysregulation of the HPA axis and childhood obesity.

In the present study, we explored the characteristics of diurnal cortisol rhythm in children and adolescents with obesity by repeated sampling of salivary cortisol over the course of a day. Moreover, we examined relationships of cortisol activity with the degree of adiposity, pubertal stage and physical activity. This information may contribute to our understanding of the associations between chronodisruption, obesity and lifestyle to provide new insight for the primary prevention of childhood obesity.

## Methods

### Participants

In this cross-sectional study, a total of 57 children and adolescents aged 6–15 years were recruited from the Department of Endocrinology and Child Health Care of Children’s Hospital of Nanjing Medical University from July 2018 to June 2019. According to WHO standards [20], the subjects were divided into a normal weight group (− 2 < BMI z-score < 1) and an obesity group (BMI z-score > 2). The exclusion criteria were as follows: (1) a history of chronic diseases (except obesity), such as epilepsy, diabetes, hypothyroidism, tumours, mental illness, precocious puberty or short stature; (2) use of exogenous steroids in the past 3 months; (3) a history of surgery, trauma or other stress events in the past 3 months; (4) use of a medication known to affect hormones; or (5) female menstrual period.

The study was approved by the Children’s Hospital of Nanjing Medical University Ethical Committee. Prior to inclusion in the study, the parents provided written informed consent.

### Measures

#### Anthropometric measures

All subjects fasted for 12 h overnight and emptied urine and stool prior to measurements. Body composition was determined using the bioelectrical impedance method (Inbody J20, Biospace, Korea), including body fat mass, fat mass percentage (FM%) and skeletal muscle mass. According to a standard protocol, height and weight were measured by experienced researchers with precisions of 0.1 cm and 0.1 kg, respectively. BMI was calculated as weight (in kilograms) divided by the square of height (in metres). Because children’s BMI varies with age and sex, BMI was converted to BMI z-score according to the World Health Organization’s Child Growth Standards (2006). Waist circumference (WC) was measured in centimetres to the nearest 0.1 cm. The waist-to-height ratio (WHtR) was calculated as WC (in centimetres) divided by height (in centimetres).

#### Pubertal stage

Professional paediatricians performed visual inspection and palpation to determine pubertal stage. Females were matched for breasts and pubic hair and males for genitalia and pubic hair [21]. The stage of pubertal development (I-V period) was assessed according to Tanner staging criteria, with Tanner II as the hallmark of puberty initiation. For analysis of different degrees of pubertal development, the Tanner stage was categorized into three levels: pre-pubertal (Tanner I), early pubertal (Tanner II and III) and late pubertal (Tanner IV and V) [21].

#### Salivary cortisol analysis

Salivary cortisol reflects the levels of biologically active, non-protein-bound cortisol in serum and follows the circadian variation in serum cortisol [22]. Salivary cortisol correlates strongly with plasma cortisol [23] and is less prone to variability due to changes in cortisol-binding proteins [24]. Due to its easy, non-invasive collection and convenient transportation and storage, salivary cortisol is widely used for paediatric research.

Salivary samples were collected at 8:00, 16:00 and 23:00 h in a quiet state after a fast of 4 h. A commercial Salivette® (SARSTEDT AG &Co, Germany) tube containing a cotton wool swab was used to collect saliva. The swab was rotated in the mouth for at least 5 min and inserted back into the tube. The cortisol samples, which are stable at room temperature for a number of days,[23] were centrifuged at 1500 rpm for 5 min within 24 h to obtain clear saliva with low viscosity, and 500 μL of saliva was pipetted into the EP tube with a micropipette. The saliva samples were stored at − 80 °C after being dispensed.

Using an Elecsys reagent kit and a Cobas e immunoassay analyser (Roche Diagnostics GmbH, Germany), cortisol levels were determined by electrochemiluminescence immunoassay (ECLIA) with a high sensitivity of 0.054 ng/ml and intra- and inter-assay coefficients of variation below 10%. Areas under the curve relative to ground (AUCs) represent the total amount of cortisol exposure during the portions of the diurnal cortisol cycle by the trapezoidal method [25]. The diurnal cortisol slope (DCS) is characterized as the decline in cortisol over the day and is calculated by the formula rise over run as the slope of the line from the first time point value to the last measured point [26]. It has been proven that there is no difference between linear regression and rise over run formulas [26]. Thus, we calculated HPA axis rhythm measures based on cortisol levels at 3 time points, AUC_08:00–16:00_, AUC_16:00–23:00_ and AUC_08:00–23:00_, as well as DCS.

#### Assessment of glucose and lipid metabolism

Blood samples were taken at 8:00 after an overnight fast of 12 h to test fasting glucose (FG), fasting insulin (FI), total cholesterol (TC), triglycerides (TG) in the obesity group and part of the normal weight group. Insulin resistance was determined by the formula of the homeostasis model assessment of insulin resistance (HOMA-IR) = ([fasting insulin (lU/mL) × fasting glucose (mmol/L)]/ 22.5.

### Questionnaires for physical activities

Children’s sleep parameters were collected by parental questionnaire. Parents reported children’s bedtime and wake-up time on weekdays and weekends during the previous month. The average sleep duration was calculated by the following formula: (sleep duration on weekdays× 5 + sleep duration on weekends× 2)/7 [27].

The Chinese Version of the Children’s Leisure Activities Study Survey questionnaire was used to assess the physical activity of the children. The questionnaire was completed by the children with the assistance of their parents, and the reliability and validity of the Chinese version has been verified [28]. A checklist of 31 physical activities and 13 sedentary behaviours was included in the questionnaire. According to the intensity of physical activity, there were 15 activities classified as vigorous-intensity physical activities (VPA, > 6 METs) and 16 activities classified as moderate-intensity (MPA, 3–5.9 METs) [28]. For data analysis, screen time consisted of 3 sedentary behaviours (SB, including watching TV or movie, playing computer games, surfing the internet or playing on the phone); the other 10 were considered non-screen sedentary behaviours.

### Statistical analyses

IBM SPSS Statistics software (Version 24.0) was used, and the level of significance was accepted with *P* < 0.05. The results are expressed as the means ± standard deviation or median [interquartile range]. The normality of data was evaluated using the Shapiro-Wilk test. Cortisol variables with a skewed distribution were logarithmically transformed for correlational analysis. Significant differences between the normal weight and obesity groups were analysed using t-tests or Mann–Whitney U-tests. Chi-square tests were applied to compare categorical variables between two groups. Differences in HPA axis measures among puberty groups were compared by analysis of variance (ANOVA) and analysis of covariance (ANCOVA). Multiple linear regressions were performed to assess the correlation of cortisol levels with different anthropometric variables and physical activities. Spearman’s correlations were employed to assess the correlation of cortisol variables with testosterone, glucose or lipid metabolism in obese children .

For analysis of 24-h movement, compositional data analysis was used following the guide of Chastin and colleagues [29]. Four compositional linear regression models were conducted for each health indicator with each behaviour sequentially entered into the model via log–ratio transformation [30]. Models examined the combined effect of the relative distribution of all movement behaviours with each health indicator [29]. Model *P* values and *R*^2^ coefficients were the same across all 4 linear regression models. Next, models assessed the association between the time spent in each movement behaviour relative to the time spent in the other movement behaviours and each health indicator. The first coefficient and its *P* value for each rotated model were used to determine whether the individual movement behaviour was significantly positively or negatively associated with each health indicator relative to the time spent in the other movement behaviours [31]. In summary, the compositional analysis is a multiple linear regression model where the cortisol measures were modelled as a function of sleep, screen time, non-screen time, and MVPA.

## Results

### Baseline characteristics

A total of 57 participants were enrolled in the study and divided into a normal weight group (*n* = 22) and an obesity group (*n* = 35) according to BMI. Demographic, anthropometric and behavioural characteristics are summarized in Table 1. There were no differences between the obesity group and the normal weight group in terms of age, sex, pubertal stage, height, sleep duration or MVPA minutes.

**Table 1:** Characteristics of study participants

Parameter	All (***n*** = 57)	Obesity (***n*** = 35)	Normal weight (***n*** = 22)	***P***
**Age (years)**	11.50 (9.35,12.85)	11.80 (10.30,13.30)	10.85 (8.98,12.13)	0.213
**Sex (boys)**	41 (72%)	26 (74%)	15 (68%)	0.618
**Pubertalstage**				
**Pre-pubertal**	19/57	11/35	8/22	0.416
**Early pubertal**	26/57	15/35	11/22	
**Late pubertal**	12/57	9/35	3/22	
**BMI z-score**	2.41 (−0.01,3.48)	3.21 (2.69,3.71)	− 0.27(− 0.88,0.35)	< 0.001
**FM (%)**				
**Boys**	36.80 (20.10,43.50)	40.15 (36.33,45.20)	12.20 (9.40,21.20)	< 0.001
**Girls**	28.85 (16.85,39.58)	38.30 (33.35,45.95)	16.70 (11.00,20.00)	< 0.001
**WHtR**	0.59 (0.41,0.63)	0.62 (0.59,0.66)	0.41 (0.38,0.42)	< 0.001
**Sleep duration (hours)**	8.93 ± 0.86	8.78 ± 0.91	9.15 ± 0.75	0.129
**MVPA (minutes)**	71.43 (40.71,106.70)	54.83 (34.67,103.80)	77.86 (53.93,139.29)	0.135
**Non-screen SB (minutes)**	240.79 ± 102.25	210.28 ± 96.88	286.56 ± 94.46	0.006
**Screen time (minutes)**	34.29 (11.40,120.00)	51.40 (12.15,128.60)	17.14 (3.21,45.21)	0.016

Data are reported as the median (interquartile range) and the Mann–Whitney U-test was used or the mean ± standard deviation and the t-test was used. *P*: obesity group vs normal weight group. *BMI z-score* body mass index z-score, *FM* fat mass, *WHtR* waist-to-height ratio, *MVPA* moderate to vigorous physical activity, *SB* sedentary behaviour

### Diurnal cortisol patterns

**Fig. 1:**
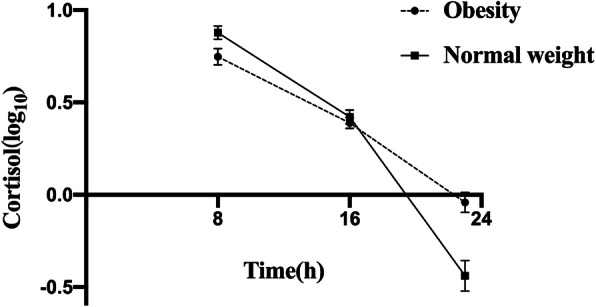
Diurnal cortisol patterns in children with obesity and normal weight. Data are expressed as the mean ± SEM, and error bars show the standard error of the mean. Cortisol variables were logarithmically transformed

Table 2 reports descriptive statistics for HPA axis rhythmicity in all subjects, which showed peak cortisol levels in the morning and a nadir at midnight. Moreover, the children with obesity displayed lower cortisol levels at 08:00 (*P* = 0.030) and AUC_08:00–16:00_ (*P* = 0.027) and higher levels at 23:00 (*P* < 0.001) than their normal weight counterparts. Figure 1 depicts the variation in the diurnal cortisol curve from 08:00 to 23:00 based on BMI category, with notably flatter trajectories of circadian cortisol observed in the children with obesity.

**Table 2:** Descriptive statistics for HPA axis rhythmicity

	All (***n*** = 57)	Obesity (***n*** = 35)	Normal weight (***n*** = 22)	***P***
**Cortisol**_**08:00**_**(nmol/L)**	6.23 (4.13,8.97)	5.79 (3.42,7.73)	8.44 (5.56,9.59)	0.030
**Cortisol**_**16:00**_ **(nmol/L)**	2.28 (1.77,3.32)	2.28 (1.72,3.18)	2.27 (1.91,3.52)	0.481
**Cortisol**_**23:00**_ **(nmol/L)**	0.59 (0.35,1.34)	1.10 (0.48,1.46)	0.40 (0.21,0.61)	< 0.001
**AUC**_**08:00–16:00**_ **(nmol/L × h)**	36.46 (25.35,48.15)	34.84 (23.01,45.99)	42.16 (32.12,52.04)	0.027
**AUC**_**16:00–23:00**_ **(nmol/L × h)**	11.73 (8.43,14.83)	11.90 (8.94,16.61)	11.51 (7.81,14.07)	0.263
**AUC**_**08:00–23:00**_ **(nmol/L × h)**	47.91 (36.31,61.56)	44.93 (34.66,60.94)	51.54 (42.75,66.87)	0.098
**DCS (nmol/L/h)**	−0.35(−0.54,-0.24)	−0.29(− 0.49,-0.14)	− 0.52(− 0.63,-0.34)	0.006

Data are reported as the median (interquartile range), and the Mann–Whitney U-test was used. *P*: obesity group vs normal weight group

### Measures of the HPA axis and pubertal stage

**Fig. 2:**
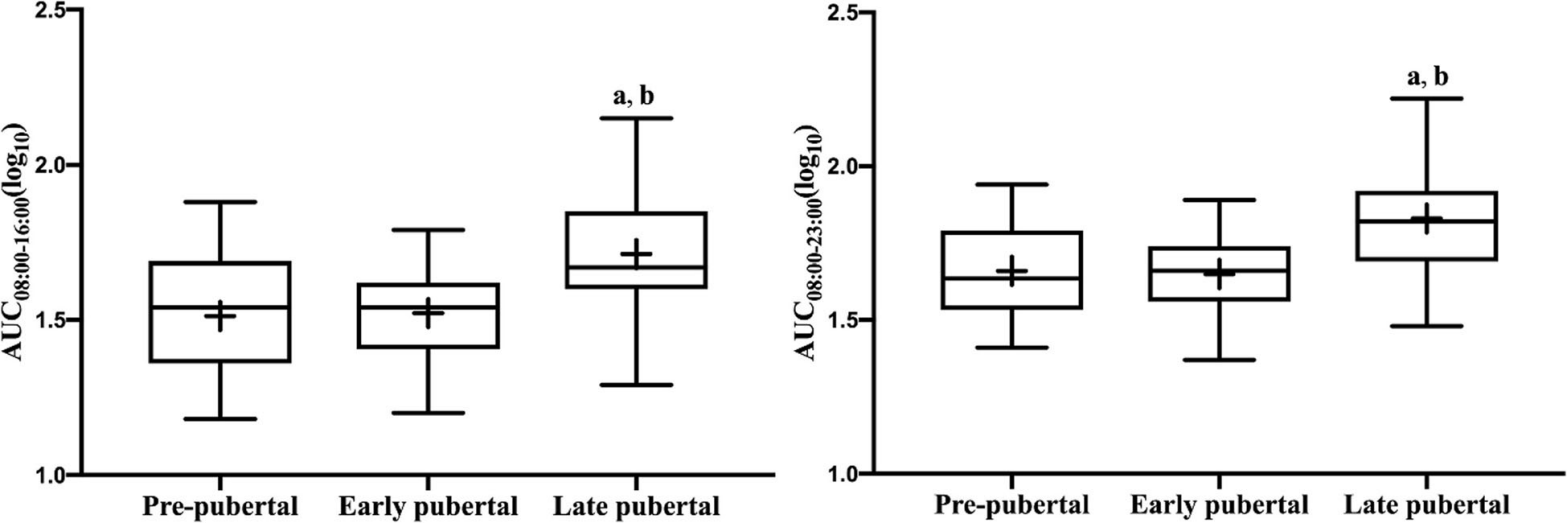
Comparison of HPA axis measures among different puberty groups. Cortisol variables were logarithmically transformed. Box plots represent interquartile range with the symbol +, inside the box plot representing the mean score. a: *P*<0.05 Pre-pubertal vs Late pubertal; b: *P*<0.05 Early pubertal vs Late pubertal

There were no significant correlations between HPA axis measures and sex or age. We then tested the hypothesis that cortisol AUC may be influenced by puberty, which was proposed in other studies [32, 33]. The AUC increased with pubertal development (AUC_08:00–16:00_:*P* = 0.008; AUC_08:00–23:00_: *P* = 0.005; ANOVA). After adjustments for BMI, the above relationships remained (AUC_08:00–16:00_: *P* = 0.002; AUC_08:00–23:00_: *P* = 0.002; ANCOVA), as shown in Fig. 2. Moreover, testosterone was positively related to AUC_08:00–16:00_ (*r* = 0.407, *P* = 0.023) and AUC_08:00–23:00_ (*r* = 0.443, *P* = 0.014) in children with obesity.

### Measures of the HPA axis and anthropometry

The results of multiple regression for associations between HPA axis measures and anthropometry in all participants are shown in Table 3. Cortisol_08:00_ was inversely associated with BMI z-score (β = − 0.247, *P* = 0.036) and WHtR (β = − 0.295, *P* = 0.027). Cortisol_23:00_ was positively associated with BMI z-score (β = 0.490, *P*<0.001), WHtR (β = 0.485, *P*<0.001) and FM% (β = 0.464, *P*<0.001), and AUC_08:00–16:00_ was inversely associated with BMI z-score (β = − 0.288, *P* = 0.033) and WHtR (β = − 0.316, *P* = 0.020). Absolute values of DCS were inversely associated with BMI z-score (β = − 0.350, *P* = 0.009), WHtR (β = − 0.384, *P* = 0.004) and FM% (β = − 0.322, *P* = 0.019). After adjustments for puberty, cortisol_08:00_ was inversely associated with BMI z-score (β = − 0.247, *P* = 0.048) and WHtR (β = − 0.271, *P* = 0.030). Cortisol_23:00_ was positively associated with BMI z-score (β = 0.454, *P*<0.001), WHtR (β = 0.484, *P*<0.001) and FM% (β = 0.451, *P*<0.001), and absolute values of DCS were inversely associated with BMI z-score (β = − 0.327, *P* = 0.013), WHtR (β = − 0.366, *P* = 0.005) and FM% (β = − 0.313, *P* = 0.017).

**Table 3:** Associations between HPA axis rhythm index and anthropometry

	BMI z-score				WHtR				FM (%)			
	β	***P***	β*	P*	β	***P***	β*	P*	β	***P***	β*	P*
**Cortisol**_**08:00**_	− 0.247	**0.036**	− 0.247	**0.048**	− 0.295	**0.027**	− 0.271	**0.030**	− 0.238	0.080	− 0.221	0.078
**Cortisol**_**16:00**_	− 0.066	0.631	− 0 .055	0.692	− 0.077	0.580	− 0.066	0.636	− 0.030	0.831	−0.016	0.907
**Cortisol**_**23:00**_	0.490	**<0.001**	0.454	**<0.001**	0.485	**<0.001**	0.484	**<0.001**	0.464	**<0.001**	0.451	**0.001**
**AUC**_**08:00–16:00**_	−0.288	**0.033**	−0.214	0.092	−0.316	**0.020**	−0.246	0.052	−0.256	0.065	−0.188	0.161
**AUC**_**16:00–23:00**_	0.157	0.265	0.192	0.161	0.188	0.266	0.192	0.162	0.181	0.205	0.229	0.092
**AUC**_**08:00–23:00**_	− 0.197	0.161	−0.123	0.349	− 0.233	0.096	−0.162	0.215	−0.170	0.234	−0.092	0.483
**|DCS|**	−0.350	**0.009**	−0.327	**0.013**	−0.384	**0.004**	−0.366	**0.005**	−0.322	**0.019**	−0.313	**0.017**

* denotes adjusted values for pubertal stage using multiple linear regression. Cortisol variables were logarithmically transformed. *BMI z-score* body mass index z-score, *FM* fat mass, *WHtR* waist-to-height ratio, *|DCS|* absolute values of diurnal cortisol slope

### HPA axis measures and 24-h physical activity

For the entire sample, correlations of each movement behaviour with HPA axis measures relative to the other movement behaviours are displayed in Table 4. After adjustments for pubertal stage and BMI z-score, inverse associations between cortisol_08:00_ (γ_MVPA_ = − 0.107; *P* = 0.018), AUC_08:00–16:00_ (γ_MVPA_ = − 0.081; *P =* 0.038), and absolute values of DCS (γ_MVPA_ = − 0.150; *P* = 0.007) with the time spent in MVPA relative to other movement behaviours were detected. Moreover, AUC_16:00–23:00_ correlated positively with time spent in non-screen sedentary behaviours (γ_non-screen SB_ = 0.169; *P* = 0.009) and negatively with the relative time spent in sleeping (γ_sleep_ = − 0.212; *P* = 0.018).

**Table 4:** Compositional behavior model for the associations between HPA axis measures and the proportion of the day spent in screen time, non-screen sedentary behaviours, MVPA, and sleep duration

Cortisol variables	Model ***P***	Model R^**2**^	γ_**Screentime**_	***P***	γ_**non-screen/SB**_	***P***	γ_**MVPA**_	***P***	γ_**sleep**_	***P***
**Cortisol**_**08:00**_	0.002	0.350	−0.004	0.895	−0.030	0.686	−0.107	**0.018**	0.142	0.183
**Cortisol**_**16:00**_	0.286	0.136	0.016	0.618	0.147	0.045	−0.009	0.377	−0.153	0.129
**Cortisol**_**23:00**_	0.004	0.332	0.004	0.941	0.224	0.080	0.086	0.246	−0.315	0.080
**AUC**_**08:00–16:00**_	0.005	0.324	0.002	0.936	0.030	0.653	−0.081	**0.038**	0.048	0.603
**AUC**_**16:00–23:00**_	0.035	0.252	0.024	0.385	0.169	**0.009**	0.019	0.594	−0.212	**0.018**
**AUC**_**08:00–23:00**_	0.009	0.309	0.010	0.711	0.064	0.292	−0.059	0.091	−0.015	0.860
**|DCS|**	0.001	0.382	−0.003	0.936	−0.069	0.445	−0.150	**0.007**	0.222	0.085

All models were adjusted for pubertal stage and BMI z-score using multiple linear regression. Cortisol variables were logarithmically transformed. Regression coefficients correspond to change in the log-ratio of the given behaviour relative to other behaviours. *MVPA* moderate to vigorous physical activity, *SB* sedentary behaviours, *|DCS|* absolute values of diurnal cortisol slope

### Measures of the HPA axis and glucose or lipid metabolism

There were no significant correlations between HPA axis measures and serum glucose or lipid levels, as shown in Table 5.

**Table 5:** Associations between HPA axis rhythm index and glucose or lipid metabolism

Cortisol variables	FG		FI		HOMA-IR		TG		TC	
	r	***P***	r	***P***	r	***P***	r	***P***	r	***P***
**Cortisol**_**08:00**_	0.144	0.345	0.009	0.955	0.011	0.944	−0.115	0.450	−0.144	0.346
**Cortisol**_**16:00**_	0.223	0.150	0.176	0.272	0.191	0.232	−0.250	0.106	−0.072	0.646
**Cortisol**_**23:00**_	0.112	0.479	0.256	0.111	0.226	0.161	0.080	0.615	−0.198	0.209
**AUC**_**08:00–16:00**_	0.223	0.152	−0.010	0.952	−0.005	0.975	−0.158	0.312	−0.124	0.429
**AUC**_**16:00–23:00**_	0.101	0.534	0.271	0.095	0.259	0.111	−0.196	0.225	−0.157	0.335
**AUC**_**08:00–23:00**_	0.216	0.180	0.132	0.424	0.120	0.467	−0.227	0.159	−0.264	0.100
**DCS**	−0.012	0.939	0.074	0.650	0.070	0.669	0.173	0.273	0.154	0.330

Spearman’s correlations and *P* values were reported. FM: fat mass, *WHtR* waist-to-height ratio, *|DCS|* absolute values of diurnal cortisol slope, *FG* fasting glucose, *FI* fasting insulin, *HOMA-IR* the homeostasis model assessment of insulin resistance, *TC* total cholesterol, *TG* triglycerides

## Discussion

In this cross-sectional study, we report the influences of obesity, puberty and physical activity on diurnal cortisol rhythm in children and adolescents. We found a dampened circadian cortisol rhythm in children with obesity, and flatter and less sharply declining slopes correlated with degrees of adiposity. The altered dynamics of the HPA axis also appeared to be influenced by puberty and the distribution of 24-h movement. Therefore, stabilizing circadian cortisol rhythms through circadian regulation strategies may be an important approach for preventing childhood obesity.

HPA axis dysfunction is a risk factor for metabolic diseases such as obesity and is closely related to negative health outcomes. It has been proven that individuals with obesity may display blunted diurnal HPA axis functioning, which mainly manifests as decreased cortisol variability, lower morning levels, or elevated evening levels [15–17]. As previously reported, obese Zucker rats lack a circadian rhythm of 11β-HSD1 gene expression in the hippocampus, which may contribute to dampened diurnal variation of circulating corticosterone levels [34]. One paediatric study demonstrated that daytime cortisol levels are inversely associated but that night-time levels are positively associated with BMI z-score and central adiposity [14]. In adults, higher BMI or WHtR correlates with a flatter diurnal cortisol slope, suggesting a shallower decline throughout the day [25, 35]. These studies incorporated multiple sampling time points, allowing more precise slope measurement and more reliable results for associations between cortisol rhythms and obesity. Similar to the above studies, we show that salivary cortisol levels were lower in the morning and higher at night with flatter and less sharply declining cortisol slopes in children with obesity than in those with a normal weight. Moreover, we found salivary cortisol slopes and night-time cortisol to be positively related to weight gain, abdominal fat distribution (WHtR) and body fat percentage in all participants. Such findings are supported by a large cross-sectional study of adults, which showed that bedtime salivary cortisol output tended to increase with BMI, indicating that individuals with obesity display abnormal HPA hyperactivity at night [36]. However, other paediatric studies have reported different findings. Based on the HPLC-MS/MS method, Chu showed higher morning salivary cortisol and morning urinary cortisol in children with obesity aged 4–5 years [37], and Kjolhede presented an inverse association between obesity and morning or evening salivary cortisol levels in children aged 6–12 years by EIA [18]. Such inconsistent findings might result from single sampling time points or different sampling times, cortisol measurements or age distributions.

Recent human studies have shown that cortisol concentrations increase significantly throughout puberty and adolescence [38, 39], which is consistent with our findings. The increased salivary cortisol AUC might reflect higher overall activity of the adrenal gland throughout puberty. In fact, the developmental process of puberty, along with endocrine changes, has been suggested to influence HPA axis functioning [40]. We also found that testosterone in children with obesity correlated positively with AUC_08:00–16:00_ and AUC_08:00–23:00_, consistent with the phenomenon of co-activation, where cortisol and testosterone (and dehydroepiandrosterone) are positively linked within an individual [41]. Accordingly, these findings highlight the important role of gonadal hormones in the development of the circadian cortisol cycle during puberty, indicating that puberty is a highly interrelated variable and should be included as a covariate in studies seeking to explore the relationship between cortisol rhythms and adolescent obesity.

Compositional analyses provide an appropriate statistical means for understanding the collective health implications of finite, co-dependent, 24-h movement behaviours [29]. In our results, the relative time spent in MVPA was related to lower morning cortisol concentrations, daytime cortisol output (AUC_08:00–16:00_) and flatter DCS, independent of puberty and BMI z-score. Labsy pointed out that acute exercise does not significantly affect steroid circadian rhythms but that medium-to-long term training, intended as chronic exercise, appeared to play a key role as a synchronizer for the whole circadian system [10]. Thus far, chronic physical activity has been reported to lower diurnal HPA activity and reduce HPA reactivity to acute stress in pre-pubertal children [42]. In cancer patients, moderate chronic PA positively influences sleep behaviour and the activity–wake circadian rhythm [43]. As lower cortisol secetion in daytime may act as a protective factor due to prior over-stimulated HPA axis in obesity [25], a reduction in morning cortisol concentrations and daytime cortisol output may also contribute to the role of MVPA as a protective factor in response to chronic stress. In both adults and children, traditional research has mainly focused on a single exercise or unclassified physical activity, without consideration of the combined effects of the composition of the rest of the day. In this study, we eliminated such drawbacks and emphasized the important role of the proportion of time spent in MVPA in the circadian system.

Our findings also suggest that increased non-screen sedentary behaviours and inadequate sleep duration are associated with higher night-time cortisol output. With sleep loss, cortisol may exert its deleterious metabolic effects by maintaining high night-time concentrations, which are associated with insulin resistance (IR), suppressed immunity and increased inflammation [44]. Thus, the findings of this integrated approach indicate that the relative distribution of time spent in different physical activities within a 24-h period is important for health promotion and maintenance of diurnal cortisol rhythm in the paediatric population.

Flat slopes with lower amplitude, i.e., those exhibiting suppressed peak levels or failing to reach sufficiently low levels by evening, are indicative of HPA dysregulation [45] and associated with a higher risk of obesity, cardiovascular disease and type 2 diabetes [17, 25]. As the circadian system also plays a role in modulating appetite with self-reported hunger peaks at night [46], elevated nadir cortisol may further increase appetite and promote the consumption of foods enriched in fat and sugar at night [13]. Moreover, fasting glucose is supposed to be lowest at night, and glucose elevation at night has been demonstrated to be temporally and quantitatively correlated with cortisol rise [47]. Human explant visceral and subcutaneous adipose tissue clock gene expression rhythms can be altered by dexamethasone administration [48]. In light of this, an interaction pathway with the HPA axis to mediate food intake and body weight via the circadian output of adipocytes is postulated [49]. In the present study, there were no associations between cortisol rhythms and glucose or lipid metabolism due to the non-corresponding sampling time to verify the above hypothesis. Nonetheless, metabolic disorders in children with obesity were increased compared with those in normal weight children (data not shown). Further analysis is warranted to verify this pattern and assess the relationship between obesity complications and cortisol slope.

Here, we present a preliminary study that examined the relationship between indices of salivary cortisol and the clinical characteristics of children and adolescents. Nonetheless, some limitations should be noted. Socioeconomic status index, dietary data and growth hormone levels were not included, and these factors may be related to cortisol rhythms. Moreover, the small sample size and sampling time limited additional findings, especially regarding verification of the correlations of cortisol rhythms with lipid and glucose metabolism. Finally, the level and intensity of PA, SB and sleep duration were parent- or self-reported, and these subjective measurements might confound the results.

## Conclusion

This study offers initial insight into the complex and interrelated associations of diurnal cortisol rhythm and obesity during childhood and adolescence. We demonstrated reduced cortisol levels in the morning and increased levels at night in childhood obesity. Flatter and less sharply declining slopes correlated with adiposity, indicating an alteration in the circadian rhythm of cortisol with adiposity. Our findings also support the importance of an appropriate distribution of 24-h movement for optimal health and the circadian system in children and young people. Synchronizing exercise and nutrient interventions to the circadian clock might maximize the health-promoting benefits of interventions to prevent and treat metabolic disease [50]. Thus, chronotherapeutic approaches targeting the maintenance of normal rhythms via a healthy lifestyle may be effective in counteracting obesity and other metabolic diseases in children and adolescents.

## Data Availability

The data used to support the findings of this study are available from the corresponding author upon request.

## Abbreviations

ANOVA: Analysis of variance
ANCOVA: Analysis of covariance
AUC: Areas under the curve
DCS: Diurnal cortisol slope
ECLIA: Electrochemiluminescence immunoassay
FM%: Fat mass percentage
FG: Fasting glucose
FI: Fasting insulin
HOMA-IR: The homeostasis model assessment of insulin resistance
MPA: Moderate-intensity physical activities
HPA axis: The hypothalamic-pituitary-adrenal axis
IR: Insulin resistance
SB: Sedentary behaviours
TC: Total cholesterol
TG: Triglycerides
VPA: Vigorous-intensity physical activities
WC: Waist circumference
WHtR: The waist-to-height ratio
